# Concordance for prognostic models with competing risks

**DOI:** 10.1093/biostatistics/kxt059

**Published:** 2014-02-02

**Authors:** Marcel Wolbers, Paul Blanche, Michael T. Koller, Jacqueline C. M. Witteman, Thomas A. Gerds

**Affiliations:** Oxford University Clinical Research Unit, Wellcome Trust Major Overseas Programme, Ho Chi Minh City, Viet Nam and Centre for Tropical Medicine, Nuffield Department of Medicine, University of Oxford, Oxford, OX3 7FZ, UK; Université Bordeaux Segalen, ISPED, INSERM U897, F-33000 Bordeaux, France; Basel Institute for Clinical Epidemiology and Biostatistics, University Hospital Basel, 4031 Basel, Switzerland; Erasmus Medical Center, 3015 Rotterdam, The Netherlands; Department of Biostatistics, University of Copenhagen, 1014 Copenhagen K, Denmark

**Keywords:** C index, Competing risks, Concordance probability, Coronary heart disease, Prognostic models, Time-dependent AUC

## Abstract

The concordance probability is a widely used measure to assess discrimination of prognostic models with binary and survival endpoints. We formally define the concordance probability for a prognostic model of the absolute risk of an event of interest in the presence of competing risks and relate it to recently proposed time-dependent area under the receiver operating characteristic curve measures. For right-censored data, we investigate inverse probability of censoring weighted (IPCW) estimates of a truncated concordance index based on a working model for the censoring distribution. We demonstrate consistency and asymptotic normality of the IPCW estimate if the working model is correctly specified and derive an explicit formula for the asymptotic variance under independent censoring. The small sample properties of the estimator are assessed in a simulation study also against misspecification of the working model. We further illustrate the methods by computing the concordance probability for a prognostic model of coronary heart disease (CHD) events in the presence of the competing risk of non-CHD death.

## 1. Introduction

Clinical decision-making and cost-effectiveness analyses often rely on prognostic models that quantify a subject's absolute risk of a disease event of interest over time. However, study populations increasingly consist of elderly individuals with varying degrees of co-morbidity who are likely to experience one of several disease endpoints other than the endpoint of main interest (Koller, Raatz *and others*, 2012). As an example, prediction of coronary heart disease (CHD) events in elderly subjects is complicated by the fact that subjects may die from other causes prior to the observation of the disease event of interest (Wolbers *and others*, 2009; Koller, Leening *and others*, 2012).

It is well known that the naive application of standard survival analysis leads to bias and risk over-estimation if competing risks are present and that specialized methods are needed (Grunkemeier *and others*, 2007; Putter *and others*, 2007). A key quantity for medical decision-making in the presence of competing risks is the absolute risk of the event of interest over time as quantified by its (covariate-dependent) cumulative incidence function (Gail and Pfeiffer, 2005; Wolbers *and others*, 2009). Thus, regression models are particularly attractive when they provide subject-specific estimates of the absolute risks based on a set of covariates (Fine and Gray, 1999; Gerds *and others*, 2012).

Several measures for quantifying the accuracy of prognostic models have been adapted from the standard survival setting with only one failure cause to competing risks. Measures include prediction error curves (Schoop *and others*, 2011), time-dependent sensitivity, specificity, and area under the receiver operating characteristic (ROC) curve (AUC) (Saha and Heagerty, 2010), and reclassification methods (Wolbers *and others*, 2009; Koller, Leening *and others*, 2012). For survival data, the concordance index (Harrell *and others*, 1982) is a frequently reported measure of discrimination and we have previously presented a simple adaptation of Harrell's concordance estimator to the competing risks setting (Wolbers *and others*, 2009).

In the present paper, we motivate and formally define a cause-specific concordance index in the presence of competing risks. Notably, the proposed concordance index depends only on the cumulative incidence function of the event of interest. We clarify the relation of the concordance to time-dependent AUC measures and discuss a possible alternative definition. We then study estimation of a truncated concordance index in the presence of right-censoring. We introduce an inverse probability of censoring weighted (IPCW) estimator and demonstrate its consistency and asymptotic normality if the working model for the censoring distribution is correctly specified. The empirical bias and mean-square error as well as coverage of asymptotic and bootstrap confidence intervals are examined in a simulation study. Finally, we illustrate the methods for an example of coronary risk prediction in older woman using data from the Rotterdam Study (Hofman *and others*, 2011).

## 2. Definition of concordance

### 2.1 Definition for a simple prognostic score without censoring

Competing risks data without censoring are given by pairs $(T,D)$ of data where $T$ is the time to the event and $D$ is the event type. For the purpose of discussing the definition and estimation of the cause-specific concordance index it is sufficient to assume that there are only two competing events. Thus, for simplicity of presentation we let $D=1$ denote the event of interest and $D=2$ the occurrence of any competing event. In applications, it may be important to model all competing events separately.

The concordance index is defined for any prognostic score $\tilde M(X)$ depending on baseline variables $X$ which can be used to order subjects with respect to the risk of an event of type 1. For example, $\tilde M(X)$ could be a single baseline marker or the linear predictor of a regression model for the event of interest derived on a training data set. We assume that higher values of $\tilde M(X)$ are associated with higher risks of the event of interest.

To motivate our definition with an example, assume that $T$ is the time to death and that a specific treatment were available which prevented death due to the event of interest ($D=1$) but would not affect death from other causes ($D=2$). The immediate benefit from such a treatment would be greatest for subject with a high risk of dying from the event of interest early, less for individuals dying from the event of interest late, and negligible for subjects with a low risk of experiencing the event of interest at all (i.e. those likely to die from competing causes). Consequently, for a random pair of subjects $(X_i,T_i,D_i)$ and $(X_j,T_j,D_j)$, the first subject would be in greater need of treatment than the second subject if they experienced the event of interest ($D_i=1$) and the second subject experienced the event of interest later ($T_i<T_j$ and $D_j=1$) or not at all ($D_j=2$). In these cases, the ranking of the risk marker for the pair of subjects is concordant if $\tilde M(X_i)>\tilde M(X_j)$. Pairs of individuals where both experience the competing event are not comparable as neither of them would be in need of treatment.

To formally define the concordance probability for the event of interest, we assume an independent test set of i.i.d. realizations of $(X_i,T_i,D_i)$ from the joint distribution of the marker and the competing risks outcome and define

(2.1) \begin{equation*}\label{eq2.1} \tilde{\mathcal C}_1=P(\tilde M(X_i)>\tilde M(X_j)\mid D_i=1 \mbox{ and } (T_i<T_j \mbox{ or } D_j=2)), \end{equation*}

for any randomly chosen pair of subjects $i,j$ from this distribution. The concordance probability for the competing event, $\tilde {\mathcal C}_2$, is defined analogously.

Define the cumulative incidence function for the event of interest as $F_1(t\mid X)=P(T\leq t,D=1\mid X)$ and the improper random variable $T^*$ as $T^*=I(D=1)\cdot T+I(D\neq 1)\cdot \infty $. $T^*$ has a distribution function equal to $F_1(t\mid X)$ for $t<\infty $ and a point mass of $1-F_1(\infty \mid X)$ at $\infty $ (Fine and Gray, 1999). As an associate editor pointed out, $\tilde {\mathcal C}_1$ can be written in terms of $T^*$ leading essentially to the standard definition of concordance for survival data: $\tilde {\mathcal C}_1=P(\tilde M(X_i)>\tilde M(X_j)\mid T_1^*<T_j^*)$.

We also note that $I(\{s<T_j \mbox { or } D_j=2\})=1-I(\{T_j\leq s, D_j=1\})$. Thus,

\[ {\rm P}\{D_i=1 \mbox{ and } (T_i<T_j \mbox{ or } D_j=2)\}= {\rm E}_{X_i,X_j}\int_0^\infty (1-F_1(s\mid X_j))\,{\rm d} F_1(s\mid X_i), \]

and we can rewrite $\tilde {\mathcal C}_1$ as

(2.2) \begin{equation*}\label{eq2.2} \tilde{\mathcal C}_1=\frac{{\mathrm{E}}_{X_i,X_j}(I(\tilde M(X_i)>\tilde M(X_j))\int_0^\infty (1-F_1(s\mid X_j)) \,{\rm d} F_1(s\mid X_i))}{{\rm E}_{X_i,X_j}(\int_0^\infty (1-F_1(s\mid X_j)) \,{\rm d} F_1(s\mid X_i))}. \end{equation*}

According to (2.2), the cause-specific concordance for event 1 depends on $F_1$ and the marginal distribution of the marker but not on the cumulative incidence function $F_2$ of the competing event. This feature is not obvious in formula (2.1) but desirable when the aim is to assess the discriminative ability of a marker for $F_1$.

In Appendix B of supplementary material available at *Biostatistics* online, we illustrate the properties of the concordance probability for a single marker and competing risks outcomes simulated according to cause-specific proportional hazards models with constant baseline hazards. The illustrations suggest that to achieve a high concordance, the marker needs to be strongly associated with an increased cause-specific hazard of the event of interest but only weakly or, even better, reversely associated with the cause-specific hazard of the competing event. This can be explained by the fact that the overall effect of a covariate on the cumulative incidence function of the event of interest depends on both cause-specific baseline hazards and both cause-specific hazard ratios (Beyersmann *and others*, 2007; Koller, Raatz *and others*, 2012).

Finally, it is important to discuss modifications of definition (2.1) for tied data (Yan and Greene, 2008). For example, it may happen that $X_i=X_j$. Depending on the application, it may then be sensible to count such pairs with a weight of $\frac 12$:

\begin{align*} \tilde{\mathcal C}_1&=P(\tilde M(X_i)>\tilde M(X_j)\mid D_i=1 \mbox{ and }(T_i<T_j \mbox{ or } D_j=2))\\ &\quad +\tfrac{1}{2}P(\tilde M(X_i)=\tilde M(X_j)\mid D_i=1 \mbox{ and }(T_i<T_j \mbox{ or } D_j=2)). \end{align*}

To simplify notation, we use definition (2.1) as the basis for our further developments.

### 2.2 An alternative definition of concordance and relation to time-dependent AUC measures

We motivated our definition of concordance with a specific treatment for the event of interest which does not affect the competing event. In this situation, a case subject $(T_i,D_i)$ with $D_i=1$ has a larger immediate benefit from treatment than a control subject $(T_j,D_j)$ with $T_j>T_i$ or $D_j=2$ as subjects experiencing a competing event have no benefit from treatment at all. However, in other situations, the treatment may affect both event types and then subjects $(T_i,D_i)$ with $D_i=1$ and $(T_j,D_j)$ with $T_j\leq T_i$ and $D_j=2$ would not be comparable. Here, it would be more relevant to distinguish cases $(T_i,D_i)$ with $D_i=1$ from those who haven not had any event up to that time point, i.e. those with $T_j>T_i$. This leads to an alternative definition of concordance:

\begin{align*} \tilde{\mathcal C}^*_{1}&=P(\tilde M(X_i)>\tilde M(X_j)\mid D_i=1 \mbox{ and }T_i<T_j)\\ &=\frac{{\mathrm{E}}_{X_i,X_j}(I(\tilde M(X_i)>\tilde M(X_j))\int_0^\infty (1-F_1(s\mid X_j)-F_2(s\mid X_j))\, {\rm d} F_1(s\mid X_i))} {{\rm E}_{X_i,X_j}(\int_0^\infty (1-F_1(s\mid X_j)-F_2(s\mid X_j))\, {\rm d} F_1(s\mid X_i))}. \end{align*}

Of note, $\tilde {\mathcal C}^*_{1}$ also depends on the cumulative incidence function of the competing event $F_2$. Thus, it might be less suitable if the main goal is to assess the relevance of a marker or a prognostic model for predicting the absolute risk of the event of interest alone, and we will not pursue it further. However, $\tilde {\mathcal C}^*_{1}$ could be valuable for assessing joint models for the cumulative incidence of both competing events.

The proposed concordance measures are closely related to measures of the time-dependent AUC which have been proposed to assess discrimination for competing risks data at a fixed time point $s$ (Saha and Heagerty, 2010; Zheng *and others*, 2012; Blanche *and others*, 2013). We review these measures in Appendix C of supplementary material available at *Biostatistics* online, and show that $\tilde {\mathcal C}_1$ can be written as a weighted average of one proposed time-dependent AUC measure over time. This is in analogy with a similar result for survival analysis without competing risks (Heagerty and Zheng, 2005) and supports the use of $\tilde {\mathcal C}_1$ as a global summary measure of performance.

### 2.3 Assessing prediction models in right-censored data

We now generalize the concordance index defined in Section 2.1 in two ways. First, we replace the simple prognostic score $\tilde M(X)$ by a more general prediction model $M(t,X)$ for the risk of event $1$ until time $t$, i.e. estimates of $F_1(t\mid X)=P(T\leq t,D=1\mid X)$, which can be obtained by combining cause-specific hazards models, by fitting a Fine and Gray regression model or by direct binomial regression (Fine and Gray, 1999; Scheike and others, 2008). Secondly, to include the typical application where individuals have a limited duration of follow-up we define a truncated version of the concordance index. Following Uno *and others* (2011) and Gerds *and others* (2013), we define

(2.3) \begin{equation*}\label{eq2.3} {\mathcal C}_{1}(t):=P(M(t,X_i)>M(t,X_j)\mid D_i=1 \mbox{ and } T_i\leq t \mbox{ and } (T_i<T_j \mbox{ or } D_j=2)). \end{equation*}

The parameter ${\mathcal C}_1(t)$ quantifies the ability of the model to correctly rank events of interest up to time $t$ and to discriminate them from competing events. The truncation is necessary to enable estimation of ${\mathcal C}_1(t)$ from right-censored data with a limited follow-up duration.

As before, we can write the truncated concordance (2.2) as a functional of $F_1$ and of the marginal distribution of the predictor values of a pair of individuals $(X_i,X_j)$: If we introduce notation for the order of the predicted risks at time $t$ for a pair of individuals,

\[ Q^{ij}(t)=I\{M(t,X_i)>M(t,X_j)\}, \]

then

(2.4) \begin{equation*}\label{eq2.4} {\mathcal C}_1(t)=\frac{{\mathrm{E}}_{X_i,X_j}(Q^{ij}(t)\int_0^t (1-F_1(s\mid X_j))\, {\rm d} F_1(s\mid X_i))}{{\rm E}_{X_i,X_j}(\int_0^t (1-F_1(s\mid X_j))\, {\rm d} F_1(s\mid X_i))}. \end{equation*}

## 3. Estimation of concordance in the presence of right-censoring

### 3.1 Right-censored data

To indicate the end of follow-up for subject $i,$ we introduce a subject-specific censoring time $C_i$. Thus, we observe only $(X_i,\tilde T_i, \tilde D_i,\Delta _i)$ $(i=1\cdots n)$ where $\tilde {T_i}=\min (T_i,C_i)$, $\Delta _i=I\{T_i\le C_i\}$ and $\tilde {D_i}=\Delta _i D_i$. We also use the following notation:

\begin{align*} \tilde{N}^1_i(t)&=I\{\tilde{T}_i\leq t, \tilde D_i=1 \}, \quad {N}^1_i(t)=I\{T_i\leq t, D_i=1 \},\\ \tilde{N}^2_i(t)&=I\{\tilde{T}_i\leq t, \tilde D_i=2 \}, \quad {N}^2_i(t)=I\{T_i\leq t, D_i=2 \},\\ \tilde{A}_{ij}&=I\{\tilde{T}_i<\tilde{T}_j\}, \quad A_{ij}=I\{T_i<T_j\},\\ \tilde{B}_{ij}&=I\{\tilde{T}_i\geq \tilde{T}_j \mbox{ and } \tilde{D_j}=2\}, \quad B_{ij}=I\{T_i\geq T_j \mbox{ and } D_j=2\}. \end{align*}

The event-free survival probability conditional on the covariate $X_i$ is then given by

\[ S(t\mid X_i)=1-{\rm E}(N_i^1(t)\mid X_i) - {\rm E}(N_i^2(t)\mid X_i)=1-F_1(t\mid X_i)-F_2(t\mid X_i). \]

We allow the censoring distribution to depend on the covariates $X_i$ but assume throughout that $C_i$ is conditionally independent of $(T_i,D_i)$ given $X_i$. This implies

(3.1) \begin{equation*}\label{eq3.1} {\mathrm{P}}(\tilde T_i>t\mid X_i)=G(t\mid X_i)S(t\mid X_i), \end{equation*}

where $G(t\mid X_i)=P(C_i>t\mid X_i)$ is the conditional probability of being uncensored at time $t$. Noting $G(t-| X_i)=P(C_i\ge t\mid X_i)$ we also have for $k=1,2$:

(3.2) \begin{equation*}\label{eq3.2} {\mathrm{E}}(\tilde N_i^k(t)\mid X_i)= {\rm E}(\Delta_i N_i^k(t)\mid X_i) = \int_0^t {\rm E}(C_i\ge s\mid X_i)\,{\rm E}({\rm d} N^k_i(s)\mid X_i) = \int_0^t G(s-|X_i)\, {\rm d} F_k(s\mid X_i). \end{equation*}

### 3.2 Ignoring non-evaluable pairs

An asymptotically biased estimate of ${\mathcal C}_1(t)$ is given by

\[ \hat{\mathcal C}_{1,{\rm naive}}(t)= \frac{\sum_{i=1}^n\sum_{j=1}^n (\tilde{A}_{ij}+\tilde{B}_{ij}) Q^{ij}(t) \tilde{N}^1_i(t)} {\sum_{i=1}^n\sum_{j=1}^n (\tilde{A}_{ij}+\tilde{B}_{ij}) \tilde{N}^1_i(t)}, \]

where $Q^{ij}(t)=I\{M(t,X_i)>M(t,X_j)\}$ is an indicator for the order of predicted risks at time $t$. This can be interpreted as the proportion of definitely concordant pairs amongst evaluable pairs, i.e. pairs for which one individual experiences the event of interest and concordance can be decided based on the observed (potentially censored) data.

This estimate evaluated at the time $t$ corresponding to the maximum follow-up duration is a direct adaptation of Harrel's ${\mathcal C}$ for survival data (Harrell *and others*, 1982) to the competing risks context and has been previously defined in Wolbers *and others* (2009). While simple, a major problem of this estimator is that by ignoring non-evaluable pairs without any correction, bias is introduced. It is well known that Harrel's ${\mathcal C}$ depends on the censoring distribution (Uno *and others*, 2011; Gerds *and others*, 2013) and $\hat {\mathcal C}_{1,{\mathrm {naive}}}(t)$ shares this limitation.

### 3.3 IPCW estimate

We derive an IPCW estimate for ${\mathcal C}_1(t)$ based on a *working model* $\mathcal G$ for $G$. Let $\tau $ be a time point where $\inf _x G(\tau \mid x)>\epsilon >0.$ We assume that the model $\mathcal G$ is correctly specified and that for all $t<\tau $ there exists a uniformly consistent, weakly asymptotically linear estimator sequence $\hat {G}$ with influence function ${\rm IF}_G(t,x;\tilde T_i,\tilde D_i,X_i)$. This implies (Bickel *and others*, 1993):

(3.3) \begin{equation*}\label{eq3.3} \sqrt n \{\hat{G}(t\mid x)-G(t\mid x)\} = \frac{1}{\sqrt n} \sum_{i=1}^n {\mathrm{IF}}_G(t,x;\tilde T_i,\tilde D_i,X_i) + o_p(1). \end{equation*}

For example, we can specify a Cox regression model and use the estimate

\[ \hat G(t\mid X_i)=\exp\left\{-\int_0^t \exp(\hat\gamma^T X_i)\hat\Gamma_0(s) \,{\rm d} s\right\}, \]

where $\hat \Gamma _0$ is the Breslow estimator of the baseline hazard function and $\hat \gamma $ the maximum partial likelihood estimator of the regression coefficients. If the censoring is conditionally independent of the competing risks outcome given the predictors and the Cox model correctly specified, then condition (3.3) is satisfied (see, e.g. Cheng *and others*, 1998). As an alternative, we could assume that the censoring is independent of the competing risks outcome and the predictors. If this assumption is correct, then the Kaplan–Meier estimate for the censoring distribution satisfies (3.3).

Based on $\hat G,$ we construct the weights

\[ \hat{W}_{ij,1}=\hat{G}(\tilde{T}_i-|X_i)\hat{G}(\tilde{T}_i|X_j) \hbox{ and } \hat{W}_{ij,2}=\hat{G}(\tilde{T}_i-|X_i)\hat{G}(\tilde{T}_j-|X_j), \]

and define an IPCW estimate of ${\mathcal C}_1(t)$:

(3.4) \begin{equation*}\label{eq3.4} \hat{\mathcal C}_{1}(t)= \frac{\sum_{i=1}^n\sum_{j=1}^n (\tilde{A}_{ij}\hat{W}_{ij,1}^{-1}+\tilde{B}_{ij}\hat{W}_{ij,2}^{-1}) Q^{ij}(t) \tilde{N}^1_i(t) } {\sum_{i=1}^n\sum_{j=1}^n (\tilde{A}_{ij}\hat{W}_{ij,1}^{-1}+\tilde{B}_{ij}\hat{W}_{ij,2}^{-1}) \tilde{N}^1_i(t)}. \end{equation*}

**Lemma 3.1**

If the working model is correctly specified and $\hat {G}$ is a consistent estimator of $G,$ then $\hat {\mathcal C}_{1}(t)$ is a consistent estimator of ${\mathcal C}_1(t)$ for all $t<\tau $. Furthermore, if (3.3) is satisfied, then $\hat {\mathcal C}_1(t)$ is asymptotically linear and $\sqrt n \{\hat {{\mathcal C}}_1(t)- {\mathcal C}_1(t)\}$ converges in distribution to a normal random variable with mean 0.

A proof of the lemma is given in Appendix D of supplementary material available at *Biostatistics* online. For the case of independent censoring, supplementary material available at *Biostatistics* online also presents an explicit formula for the influence function and a consistent estimator of the asymptotic variance. The proposed concordance estimator has been implemented with the function cindex of the R package pec (Mogensen *and others*, 2012). Example code is provided in Appendix A of supplementary material available at *Biostatistics* online.

## 4. Simulation study

A simulation study was performed to assess bias and root mean square error (RMSE) of the proposed IPCW estimator and coverage of asymptotic and bootstrap confidence intervals. Simulations were for a single prognostic marker $X$ and a parameter-free time-independent model $M(X,t)=X$. The covariate $X$ was simulated to follow a standard normal distribution.

Conditional on $X$, uncensored competing risks data $(T,D)$ was assumed to follow cause-specific Cox-exponential models (Bender *and others*, 2006):

(4.1) \begin{equation*}\label{eq4.1} \begin{split} \text{Event 1: }\lambda_1(t\mid X)&=\lambda_{01}\exp(\beta_1 X),\\ \text{Event 2: }\lambda_2(t\mid X)&=\lambda_{02}\exp(\beta_2 X). \end{split} \end{equation*}

This was implemented by simulating latent exponentially distributed event times $T_1$ and $T_2$ and then setting $T=\min (T_1,T_2)$ and $D=1$ for $T_1<T_2$ and $D=2$ for $T_1\ge T_2$. We consider two competing risks scenarios. In scenario CR1, we set $\lambda _{01}(t)=1, \lambda _{02}(t)=2,$ and $\beta _1= \beta _2=1$. In scenario CR2, we set $\lambda _{01}(t)=1, \lambda _{02}(t)=0.5,$ and $\beta _1= 2,\beta _2=-1$. As truncation time points, we used the median and the 75% quantile $q_{75}$ of the marginal distribution of $T$. The truncation time points and corresponding true values of ${\mathcal C}_1(t)$ were determined by simulation based on a large uncensored data set of size $100\,000$.

Censoring times $C$ were drawn under a third Cox-exponential model:

\[ \lambda_{{\rm cens}}(t\mid X)=\lambda_{0,{\rm cens}}\cdot\exp(\gamma_1 X). \]

The observed time $\tilde T$ was obtained as the minimum of $T$ and $C$ and considered as right-censored if $C<T$. We repeated the simulations for independent censoring ($\gamma _1=0$) and covariate-dependent censoring ($\gamma _1=1$). For each truncation time point $t,$ the values of $\lambda _{0,{\mathrm {cens}}}$ were found by simulation such that the expected proportion of right-censored event times amongst observations with $\tilde T<t$ was 25%, 50%, or 75%, respectively. For each of the scenarios, we report results for sample sizes 250 and 1000 averaged across 1000 simulated data sets.

In each simulated data set, we computed three different estimators of ${\mathcal C}_1(t)$: the naive estimator $\hat {{\mathcal C}}_{1,{\mathrm {naive}}}(t)$, the IPCW estimator based on the marginal Kaplan–Meier estimate of the censoring distribution $\hat {{\mathcal C}}_{1,{\mathrm {KM}}}(t)$ and the IPCW estimator based on a Cox regression model for the censoring distribution $\hat {{\mathcal C}}_{1,{\rm Cox}}(t)$.

Bias and root mean squared errors for $t$ chosen as the $75\%$-quantile of $T$ are shown in Table 1. Table 2 shows the associated coverage of percentile bootstrap (all estimators) and asymptotic Wald-type confidence intervals ($\hat {{\mathcal C}}_{1,{\rm KM}}(t)$ only) and contrasts empirical standard errors with average bootstrap and asymptotic standard errors for the estimate $\hat {{\mathcal C}}_{1,{\mathrm {KM}}}(t)$. Corresponding results for $t$ chosen as the median are shown in Appendix E of supplementary material available at *Biostatistics* online.

**Table 1.** Average bias and RMSE for $3$ different estimators of ${\mathcal C}_1(t)$ averaged over $1000$ data sets simulated under the $2$ scenarios CR1 and CR2 for varying sample size $N,$ independent $(\gamma _1=0),$ or covariate-dependent censoring $(\gamma _1=1),$ respectively$,$ and varying censoring rates

$N$	$\gamma _1$	Censored before $t$ (%)	$\hat {{\mathcal C}}_{1,{\mathrm {naive}}}(t)$	$\hat {{\mathcal C}}_{1,{\mathrm {KM}}}(t)$	$\hat {{\mathcal C}}_{1,{\mathrm {Cox}}}(t)$
CR1: ${\mathcal C}_1(t)=62.1\%, \beta _1=1, \beta _2=1, \lambda _{01}=1, \lambda _{02}=2$, $t= q_{75}$					
250	0	25	1.5 (4.0)	0.1 (3.7)	0.1 (3.7)
250	0	50	3.9 (5.9)	0.3 (4.8)	0.3 (4.8)
250	0	75	8.1 (10.0)	2.6 (10.1)	2.8 (9.5)
250	1	25	1.0 (4.0)	$-$0.1 (3.9)	0.1 (3.9)
250	1	50	2.6 (5.4)	$-$0.3 (4.9)	$-$0.2 (5.8)
250	1	75	5.1 (8.4)	0.2 (8.2)	$-$1.9 (10.3)
1000	0	25	1.4 (2.3)	0.0 (1.9)	0.0 (1.9)
1000	0	50	3.8 (4.3)	0.0 (2.3)	0.0 (2.3)
1000	0	75	7.9 (8.4)	1.0 (6.4)	1.2 (6.0)
1000	1	25	0.9 (2.1)	$-$0.3 (1.9)	0.0 (2.0)
1000	1	50	2.5 (3.3)	$-$0.5 (2.3)	$-$0.1 (3.4)
1000	1	75	5.0 (5.9)	$-$0.4 (3.8)	$-$1.8 (7.5)
CR2: ${\mathcal C}_1(t)=85.0\%, \beta _1=2, \beta _2=-1, \lambda _{01}=1, \lambda _{02}=0.5$, $t= q_{75}$					
250	0	25	0.7 (1.8)	0.1 (1.7)	0.1 (1.7)
250	0	50	1.5 (2.4)	0.2 (2.1)	0.2 (2.1)
250	0	75	2.8 (3.7)	0.7 (4.1)	0.8 (3.7)
250	1	25	0.6 (1.9)	0.0 (1.8)	0.1 (1.7)
250	1	50	1.3 (2.5)	$-$0.1 (2.3)	0.1 (2.3)
250	1	75	2.2 (3.8)	$-$0.2 (4.4)	$-$0.3 (5.6)
1000	0	25	0.7 (1.1)	0.0 (0.8)	0.0 (0.8)
1000	0	50	1.4 (1.7)	0.0 (1.0)	0.0 (1.0)
1000	0	75	2.7 (3.0)	0.2 (2.8)	0.3 (2.5)
1000	1	25	0.6 (1.0)	0.0 (0.9)	0.1 (0.8)
1000	1	50	1.3 (1.6)	$-$0.2 (1.2)	0.1 (1.1)
1000	1	75	2.1 (2.6)	$-$0.7 (2.4)	$-$0.7 (4.3)

$t$ was chosen as the $75$-quantile of the marginal time-to-event distribution. Column $3$ shows the expected proportion of right-censored event times amongst observations with $\tilde T<t$. Columns $4$–$6$ show average bias $($RMSE$)$ for the three estimators $($multiplied by $100$ for easier readability$)$.

**Table 2.** Coverage of confidence intervals for the same simulation scenarios as in Table 1

			Standard error KM			Coverage			
$N$	$\gamma _1$	Censored before $t$ (%)	Empirical	Average asymptotic	Average bootstrap	Asymptotic KM	Bootstrap naive	Bootstrap KM	Cox
CR1: ${\mathcal C}_1(t)=62.1\%, \beta _1=1, \beta _2=1, \lambda _{01}=1, \lambda _{02}=2$, $t= q_{75}$									
250	0	25	0.0372	0.0384	0.0384	94.5	91.6	94.2	94.3
250	0	50	0.048	0.0473	0.0478	93.4	83.6	93.6	93.2
250	0	75	0.0974	0.0707	0.077	77.8	67.3	81.2	82.1
250	1	25	0.0388	0.0392	0.0395	94.2	93.4	94.5	95.1
250	1	50	0.0486	0.0479	0.0486	93.8	90.1	94.1	93.5
250	1	75	0.0823	0.0696	0.0733	86.4	81.4	88.6	88.4
1000	0	25	0.0188	0.0192	0.019	95.4	88.9	95.1	95.2
1000	0	50	0.0234	0.0238	0.0238	95.1	59.6	95.3	95.1
1000	0	75	0.0635	0.0475	0.0499	80.7	23.2	81.9	82.6
1000	1	25	0.0193	0.0195	0.0195	94.9	91.6	94.6	94.3
1000	1	50	0.0229	0.0239	0.0239	95.4	81.6	95.5	93.4
1000	1	75	0.0376	0.0368	0.037	93.9	64.6	93.8	89.2
CR2: ${\mathcal C}_1(t)=85.0\%, \beta _1=2, \beta _2=-1, \lambda _{01}=1, \lambda _{02}=0.5$, $t= q_{75}$									
250	0	25	0.0168	0.0173	0.0174	95.1	91.4	95.0	95.0
250	0	50	0.0207	0.0208	0.021	94.3	84.2	94.5	94.4
250	0	75	0.0404	0.0343	0.0359	85.4	72.5	88.2	87.9
250	1	25	0.0181	0.0183	0.0186	95.0	92.2	95.3	95.2
250	1	50	0.0232	0.0235	0.024	94.6	87.9	95.2	95.9
250	1	75	0.0439	0.0384	0.0399	87.4	81.2	88.8	93.3
1000	0	25	0.00831	0.00859	0.00856	95.3	86.7	95.5	95.6
1000	0	50	0.0101	0.0103	0.0103	95.6	66.7	95.4	95.7
1000	0	75	0.0277	0.0218	0.0224	87.4	41.4	88.8	89.8
1000	1	25	0.00878	0.00905	0.00914	95.2	88.7	95.4	95.9
1000	1	50	0.0116	0.0118	0.0119	95.8	76.5	95.9	95.3
1000	1	75	0.0234	0.022	0.0221	93.7	67.6	94.2	94.0

Columns $8$–$10$ display observed coverage of $95\%$ percentile bootstrap confidence intervals for all three estimators$,$ column $7$ shows coverage of asymptotic Wald-type confidence intervals for $\hat {{\mathcal C}}_{1,{\mathrm {KM}}}(t)$. Columns $4$–$6$ show the empirical standard error for the $1000$ estimates and the average asymptotic and bootstrap standard errors of $\hat {{\mathcal C}}_{1,{\mathrm {KM}}}(t)$.

Based on Tables 1 and 2, we draw the following conclusions.

1. The naive estimator $\hat {{\mathcal C}}_{1,{\mathrm {naive}}}(t)$ can be biased and this can lead to insufficient coverage.
2. The ICPW estimator $\hat {{\mathcal C}}_{1,{\mathrm {KM}}}(t)$ can also be biased if the censoring depends on the covariate. In some cases, $\hat {{\mathcal C}}_{1,{\mathrm {Cox}}}(t)$ has a smaller bias but for high rates of censoring it can do worse than $\hat {{\mathcal C}}_{1,{\mathrm {KM}}}(t)$ even though the censoring depends on the covariate.
3. Coverage of confidence intervals for $\hat {{\mathcal C}}_{1,{\mathrm {KM}}}(t)$ and $\hat {{\mathcal C}}_{1,{\mathrm {Cox}}}(t)$ was generally close to the nominal 95% except for some scenarios with high censoring rates of 75%. Both average asymptotic and bootstrap standard errors closely resembled the empirical standard errors of $\hat {{\mathcal C}}_{1,{\mathrm {KM}}}(t)$.

## 5. Application to coronary risk prediction

Specialist medical societies recommend initiation of preventive treatment for CHD based on a subjects’ predicted 10-year risk for CHD (NCEP, 2002). To accurately predict the absolute risk of CHD in older people, prognostic models for CHD need to account for the competing risk of non-CHD death (Koller, Leening *and others*, 2012). In this section, we revisit the example of Wolbers *and others* (2009) on coronary risk prediction based on data of elderly women from the Rotterdam Study, a prospective, population-based cohort of elderly subjects living in a suburb area of Rotterdam, the Netherlands (Hofman *and others*, 2011).

We analyzed data from 10 years of follow-up of 4144 women aged between 55 and 90 years who were free of CHD at baseline. During that follow-up period, 389 women experienced a CHD event and 921 women died without prior CHD event. Only 41 women of those event-free had less than 10 years of follow-up. We randomly split the data set into a training data set (2763 women with 249 CHD events) and a validation data set (1381 women with 140 CHD events).

Using the training set, we estimated the parameters of a Fine–Gray regression model which included the “traditional” baseline risk factors for CHD: age, treatment for high blood pressure (yes versus no), systolic blood pressure (separate slopes depending on whether the subject was on blood pressure treatment or not), diabetes mellitus, log-transformed total cholesterol to HDL cholesterol ratio, and smoking status (current versus never or former smoker). All these risk factors were associated with an increased CHD risk and, except for diabetes, all reached conventional significance ($p<0.05$). We also investigated the role of age, the strongest predictor variable, as a simple marker for CHD.

Concordance estimates were obtained for these models in the validation set. The dependence of the censoring distribution on the covariates was investigated with a Cox regression model which yielded no trends and non-significant Wald tests for all variables. Thus, all IPCW estimates of concordance were based on the marginal Kaplan–Meier estimator for the censoring distribution from the validation set.

**Fig. 1.**
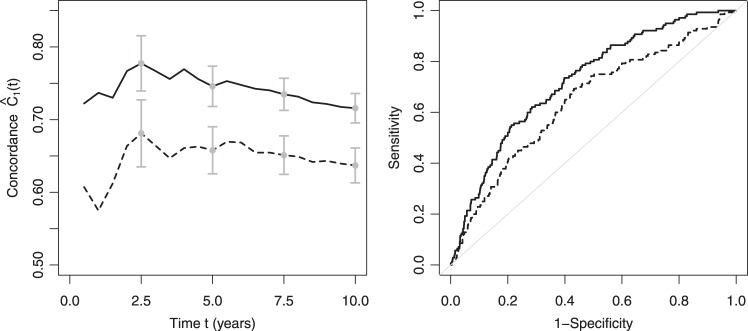
Left panel: IPCW estimates of ${\mathcal C}_1(t)$ for the multiple Fine and Gray model (solid line) and the model with age as the only covariate (dashed line) for a follow-up duration of 1–10 years in the validation data. Error bars at 2.5, 5, 7.5, and 10 years of follow-up correspond to bootstrap standard errors. Right panel: time-dependent receiver operating characteristic curve at time $t=10$ years for the multiple Fine and Gray model (solid line) and the model with age as the only covariate (dashed line) in the validation data. Cases were defined as subjects with $T\leq t$ and $D = 1$, controls as subjects with $T>t$ or $D=2$.

The left panel of Figure 1 shows the discrimination ability of the two Fine–Gray models for varying time horizons between 1 and 10 years. The model including all risk factors shows higher discriminative ability compared with the model based on age alone. For both models, concordance estimates stabilize after about 2.5 years of follow-up and remain fairly stable though slightly decreasing. The decrease may occur because earlier events are easier to predict than later events.

The right panel of Figure 1 shows time-dependent ROC curves at time $t=$10 years for the two models. For this graph, cases were defined as subjects with $T\leq t$ and $D = 1$, controls as subjects with $T>t$ or $D=2$. Estimation was also based on IPCW-weighting as implemented in the R package $timeROC$ (Blanche *and others*, 2013).

Table 3 shows the estimated concordance for predicting CHD and non-CHD death, respectively, during the 10 years follow-up in the validation data. The Fine and Gray model for non-CHD death used the same covariates as the model for CHD. Age alone is a strong predictor for non-CHD death in this elderly population but the multiple Fine and Gray model did not substantially improve concordance. This is not surprising as most additional covariates are established CHD-specific risk factors which would not be expected to strongly affect non-CHD death (except for other deaths related to the cardiovascular system).

**Table 3.** Estimated concordance and AUC measures in the validation data of the CHD study for both competing risks $($in $\%)$

	CHD			Non-CHD death		
	${\mathcal C}_1(t)$	${\mathrm {AUC}}(t)_{1,C1}$	${\mathrm {AUC}}(t)_{1,C2}$	${\mathcal C}_2(t)$	${\mathrm {AUC}}(t)_{2,C1}$	${\mathrm {AUC}}(t)_{2,C2}$
Age only	63.7	72.3	64.3	75.7	80.8	78.0
Fine–Gray	71.6	79.4	72.4	76.2	81.3	78.6

Truncation point for concordance is $t=10$ years. AUC measures are also reported at $t=10$ years. Cumulative cases were defined for both AUC measures as subjects with $T\leq t$ and *D* = “event type studied” (CHD or non-CHD death, respectively), controls as subjects with $T>t$ for ${\rm AUC}(t)_{\cdot ,C1}$ and as subjects with $T>t$ or *D* = “other event type” for ${\mathrm {AUC}}(t)_{\cdot ,C2}$.

Table 3 also displays time-dependent AUC measures at $t=10$ years in the validation data using two different definitions of controls (Blanche *and others*, 2013). AUCs with controls defined as subjects with $T>t$ or $D=\text {``other event type''}$ (consistently with our concordance definition) were slightly higher but comparable with the concordance whereas AUCs using only subjects with $T>t$ as controls were substantially higher. This could be explained by the fact that age is a strong predictor of both CHD and non-CHD death which hampers discrimination of CHD events from non-CHD deaths.

## 6. Discussion

We have presented a formal definition of the concordance probability for prognostic models in the presence of competing risks. Like the concordance probability for survival or binary data, it provides a simple overall numeric measure of discrimination. To deal with right-censored data, we derived an IPCW estimator of the truncated concordance probability and established consistency and asymptotic normality under mild assumptions. Asymptotic properties of the proposed estimator rely on the assumption that the censoring distribution is correctly specified and conditionally independent of the competing risks process given covariates. In many applications, it will be reasonable to assume that the censoring mechanism does not depend on covariates and then the marginal Kaplan–Meier estimate of the censoring distribution can be used. However, if for some reason the design or conduct of a clinical study introduced a dependence between the follow-up duration and covariates which also affect the competing risks process (any component, including the cause-specific hazards of competing events), then it is recommended to use a working regression model for the censoring distribution in order to avoid biased conclusions.

The fact that we estimate a truncated version of concordance rather than the unconstrained concordance probability should not be seen as a limitation of our approach. Indeed it is impossible to assess the performance of prognostic models beyond the maximum follow-up duration without strong and untestable assumptions. If we assume independent censoring, our estimator is defined if we truncate at any time before or at the largest observed censoring or event time. As for the Kaplan–Meier estimator, the effect of censoring on the variability of the IPCW estimator is increasing with increasing truncation time. However, it is difficult to recommend a general purpose truncation time, in particular because the truncation time point influences the interpretation of the concordance probability. To avoid unstable results in practical applications, we recommend that the analyst develops an appropriate model for the censoring distribution, e.g. the Kaplan–Meier estimator or a Cox model, and then investigates the predicted probabilities of being uncensored at the candidate truncation times. Multiple truncation time points can be evaluated and it can be useful to compare discrimination ability at different truncation time points. A model which is good at discriminating patients with an early failure time (e.g. after surgery) from others may not be good at discriminating subsequent failure times amongst patients who survive a first high risk period.

As discussed in Appendix C of supplementary material available at *Biostatistics* online, our approach is related to time-dependent sensitivity, specificity, and ROC curves for competing risks (Saha and Heagerty, 2010) and the concordance can be written as a weighted average of the time-dependent ${\mathrm {AUC}}(s)$ for incident cases ($T=s$, $D=1$) and controls defined as observations with $T>s$ or $D=2$. Thus, the concordance serves as an overall summary of discrimination whereas the time-dependent AUC measures discrimination of the event status at one specific time point.

We assumed that the prognostic model was derived on an independent training data set and only in this setting are asymptotic or bootstrap confidence intervals for the truncated concordance readily available. Clearly, independent training data are not always available, and even if they are, a joint analysis of all data will be more efficient. However, some form of internal cross-validation is needed to develop and assess a prognostic model with a single data set (Efron and Tibshirani, 1997; Gerds *and others*, 2008; Hastie *and others*, 2009).

It is important to emphasize that our definition of concordance assesses a prognostic model for the absolute risk of the event of interest in the presence of competing risks. In line with earlier work (Gail and Pfeiffer, 2005; Wolbers *and others*, 2009), we regard this risk as crucial for medical decision-making in the competing risks setting. However, in many instances explicit consideration of competing events will also be important and modeling the entire competing risks multi-state process will provide further insights (Beyersmann *and others*, 2007). As an example, our illustration of concordance for a single marker (presented in supplementary material available at *Biostatistics* online) shows that discrimination of prognostic models for the event of interest is hampered if covariates affect both cause-specific hazards with regression coefficients of the same sign, especially if there is a strong association with the competing risk or if the baseline competing hazard is high. This indicates that to achieve high discrimination ability one needs predictors which are only weakly or, even better, reversely associated with the cause-specific hazard of the competing event. Moreover, in settings where all competing events are of similar importance, joint accuracy criteria for the entire competing risks multi-state process are needed and their development is an important area for future research.

## Supplementary material

Supplementary material is available at http://biostatistics.oxfordjournals.org.

## Funding

M.W. was supported by the Wellcome Trust and the Li Ka Shing Foundation—University of Oxford Global Health Programme. Funding to pay the Open Access publication charges for this article was provided by the Wellcome Trust.

## Supplementary Material

Supplementary Data
