# Supplementary material for: Concordance for prognostic models with competing risks
Source: Biostatistics. 2014 Feb 2;15(3):526–39. doi: 10.1093/biostatistics/kxt059 (PMC4059461; doi:10.1093/biostatistics/kxt059)
Supplement: Supplementary Data [file supp_15_3_526__index.html]

Concordance for prognostic models with competing risks — Supplementary Data 

# Concordance for prognostic models with competing risks

## Supplementary Data

Supplementary Data

**Files in this Supplementary Material:**

- Supplementary Data - Pdf file
