# Supplementary material for: Concordance for prognostic models with competing risks
Source: Biostatistics. 2014 Feb 2;15(3):526–39. doi: 10.1093/biostatistics/kxt059 (PMC4059461; doi:10.1093/biostatistics/kxt059)
Supplement: Supplementary Data [file supp_kxt059_kxt059supp_data.pdf]

Biostatistics (2014), **0**, 0, *pp.* 1–14
doi:10.1093/biostatistics/concordanceCR˙supplements˙4Feb2014

Supplementary material to "Concordance for prognostic models with competing risks"

MARCEL WOLBERS*
Oxford University Clinical Research Unit, Wellcome Trust Major Overseas Programme, Ho Chi Minh City, Viet Nam, and Centre for Tropical Medicine, Nuffield Department of Medicine, University of Oxford, Oxford, OX3 7FZ, UK

mwolbers@oucru.org

PAUL BLANCHE

Université Bordeaux Segalen, ISPED, INSERM U897, F-33000 Bordeaux, France

MICHAEL T. KOLLER

Basel Institute for Clinical Epidemiology and Biostatistics, University Hospital Basel, 4031 Basel, Switzerland

JACQUELINE C.M. WITTEMAN

Erasmus Medical Center, 3015 Rotterdam, The Netherlands

THOMAS A. GERDS

Department of Biostatistics, University of Copenhagen, 1014 Copenhagen K, Denmark

*To whom correspondence should be addressed.

© The Author 2014. Published by Oxford University Press. All rights reserved. For permissions, please e-mail: journals.permissions@oup.com

APPENDIX

A. Software implementation

The presented concordance estimators for competing risks have been implemented in the function *cindex* of the R package *pec* (Mogensen *and others*, 2012). The following box shows R code for evaluating $\hat{\mathcal{C}}_1(t)$ at $t = 1, 5,$ and 10 simultaneously for a Fine-Gray regression model and the combination of cause-specific Cox regression with age as the only covariate based on a marginal Kaplan-Meier model for the censoring distribution.

```r
library(pec)
library(riskRegression)
library(cmprsk)

# Fit Fine-Gray and cause-specific models for failure cause 1 to the training data

fg.train <- FGR(Hist(ttevent,evtype)~age,data=chd.train,cause=1)
cox.train <- CSC(Hist(ttevent,evtype)~age,data=chd.train,cause=1)

# Calculate truncated concordance at t=1,5,10 years
# for CHD in the test data

cindex(list(fg.train,cox.train),
      formula=Hist(ttevent,evtype)~1,
      cens.model="marginal",
      data=chd.test,eval.times=c(1,5,10),cause=1)

# Fit Fine-Gray and cause-specific models for failure cause 1 in all data

fg <- FGR(Hist(ttevent,evtype)~age,data=chd,cause=1)
cox <- CSC(Hist(ttevent,evtype)~age,data=chd,cause=1)

# Calculate truncated concordance at t=1,5,10 years
# for CHD using bootstrap cross-validation

cindex(list(fg,cox),
      formula=Hist(ttevent,evtype)~1,
      cens.model="marginal",
      splitMethod="bootcv",
      B=1000,
      data=chd,eval.times=c(1,5,10),cause=1)
```

B. Illustration: the concordance probability of a continuous marker for cause-specific hazards models

We investigated the behaviour of the untruncated concordance probability $\tilde{\mathcal{C}}_1$ in the case of a time-independent prognostic marker X and further assumed a parameter-free model $\tilde{M}(X) = X$. We first generated $n = 10,000$ independent marker values X by repeatedly drawing from the standard normal distribution.

Conditional on X, uncensored competing risks data (T, D) was assumed to follow cause-specific Cox-exponential models (Bender *and others*, 2005):

$$\begin{aligned} &\text{Event 1: } \lambda_1(t|X) = \lambda_{01}\exp(\beta_1 X) \\ &\text{Event 2: } \lambda_2(t|X) = \lambda_{02}\exp(\beta_2 X). \end{aligned} \tag{B.1}$$

This was implemented by simulating latent exponentially distributed event times T_1 and T_2 and then setting $T = \min(T_1, T_2)$ and $D = 1$ for $T_1 < T_2$ and $D = 2$ for $T_1 \geqslant T_2$.

Four different scenarios were considered: (1) $\beta_2 = 0$, (2) $\beta_2 = -\beta_1$, (3) $\beta_2 = \beta_1$, (4) $\beta_2 = 2\beta_1$. We further set $\lambda_{01} = 1$ and consider three values for $\lambda_{02} = \{0, 0.5, 2\}$ where $\lambda_{02} = 0$ corresponds to no competing risks. In each scenario, we compute the untruncated concordance index $\tilde{\mathcal{C}}_1$ in a single data set of size $n = 10,000$ obtained for varying values of β_1 between 0 and 10. Results are shown in Figure 1.

A comparison of scenario (1) between the panels of figure 1 (black solid lines) shows only small changes in concordance index due to the presence of a competing risk. In scenario (2) where the covariate is associated with an increased hazard of the event of interest but a decreased hazard of the competing event, the discrimination ability is increased (black dashed lines). In contrast, the discrimination ability is markedly reduced when the marker affects both cause-specific hazards with regression coefficients of the same sign (scenarios (3) and (4); gray solid and dashed lines).

These behaviors of the concordance probability for the event of interest can be explained by the fact that the overall effect of a covariate on the cumulative incidence function of the

Fig. 1. Concordance for cause-specific hazards models depending on the effect size β_1 as well as on the baseline hazard $\lambda_{02}(t)$ and the regression coefficient for the competing risk. Black solid lines refer to $\beta_2 = 0$, black dashed lines to $\beta_2 = -\beta_1$, gray solid lines to $\beta_2 = \beta_1$, and gray dashed lines to $\beta_2 = 2\beta_1$.

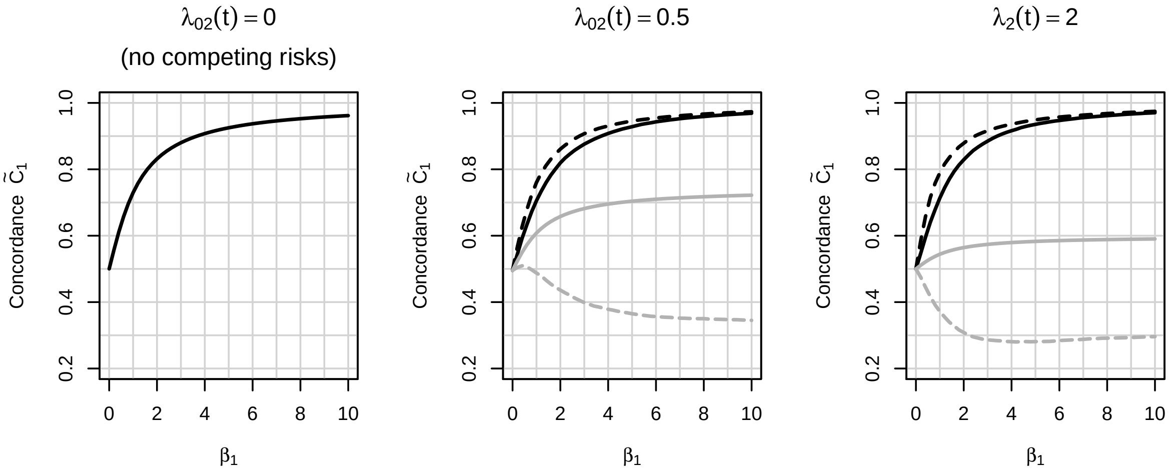

event of interest depends on both cause-specific baseline hazards and both cause-specific hazard ratios (Beyersmann *and others*, 2007; Koller *and others*, 2012). In particular, a covariate that is positively associated with the cause-specific hazard of the event of interest can simultaneously be negatively associated with the corresponding cumulative incidence function of that event if the cause-specific hazard of the competing event is larger or if it shows a stronger positive association with the covariate. This explains that the concordance index drops below 0.5 in scenario (4). Importantly, these findings suggest that the discrimination accuracy of a prognostic model for the absolute risk of the event of interest improves when there are risk factors for the event of interest which are only weakly or, even better, reversely associated with the cause-specific hazard of the competing event.

C. Time-dependent AUC measures for competing risks

Several measures for the area under the time-dependent ROC curve (AUC) have been proposed in the literature (Saha and Heagerty, 2010; Zheng *and others*, 2012; Blanche *and others*, 2013). These measures assess prognoses for the event status at a fixed follow-up time-point s whereas the concordance probability assesses prognoses for the order of the event times. For assessing accuracy of the event of interest, two different case and control definitions, respectively, have been proposed:

Cumulative cases (C):	$T \leqslant s, D = 1$	Incident cases (I):	$T = s, D = 1$
Control (definition 1):	$T > s$	Control (definition 2):	$T > s$ or $D = 2$

(Saha and Heagerty, 2010) note that cumulative cases are important when the main interest is in using baseline markers to distinguish between those who will fail from the event of interest before time s from those who do not. Incident cases are important when the interest is in distinguishing cases with an event at time s among those remaining at risk.

Control definition 1 considers those as controls who are at risk beyond time s using a standard risk set definition whereas for definition 2, subjects experiencing competing event remain evaluable as controls indefinitely (Fine and Gray, 1999). To motivate our definition of concordance in Section 2.1, we looked at the example of evaluating the benefit of a specific treatment for the event of interest which does not affect the competing event and argued that (cumulative and incident) cases would have a more immediate need for treatment than controls according to definition 2. In particular, subjects experiencing a competing event have no benefit from treatment at all. In this situation, the control definition 2 would be most relevant. However, in other situations, a treatment might affect both event types, and it would be most relevant to distinguish cases from those who haven't had any event up to that time point, i.e. to use control definition 1.

For incident cases and the second definition of controls (I2), the AUC for the event of interest

is defined as

$$\begin{aligned} AUC_{1,I2}(s) &= P(\tilde{M}(X_i) > \tilde{M}(X_j) | D_i = 1 \text{ and } T_i = s \text{ and } (T_j > s \text{ or } D_j = 2)) \\ &= \frac{\mathrm{E}_{X_i,X_j}(I(\tilde{M}(X_i) > \tilde{M}(X_j))(1 - F_1(s|X_j))f_1(s|X_i))}{\mathrm{E}_{X_i,X_j}((1 - F_1(s|X_j))f_1(s|X_i))} \end{aligned}$$

with $\mathrm{d}F_1(s|X) = f_1(s|X)ds$. By comparing the above formula with (2.2) in the main text, it is easy to see that the concordance $\tilde{\mathcal{C}}_1$ is a weighted average of the $AUC_{1,I2}(s)$ over time:

$$\tilde{\mathcal{C}}_1 = \int_0^\infty AUC_{1,I2}(s)w(s)ds \text{ with weights } w(s) = \frac{\mathrm{E}_{X_i,X_j}((1 - F_1(s|X_j))f_1(s|X_i))}{\mathrm{E}_{X_i,X_j}(\int_0^\infty (1 - F_1(s|X_j))\mathrm{d}F_1(s|X_i))}$$

This is in analogy with a similar result for survival analyis (Heagerty and Zheng, 2005).

Similarly, (Zheng *and others*, 2012; Blanche *and others*, 2013) defined an $AUC(s)$ for cumulative cases and definition 2 of controls (C2):

$$\begin{aligned} AUC_{1,C2}(s) &= P(\tilde{M}(X_i) > \tilde{M}(X_j) | D_i = 1 \text{ and } T_i \leqslant s \text{ and } (T_j > s \text{ or } D_j = 2)) \\ &= \frac{\mathrm{E}_{X_i,X_j}(I(\tilde{M}(X_i) > \tilde{M}(X_j))(1 - F_1(s|X_j))F_1(s|X_i))}{\mathrm{E}_{X_i,X_j}((1 - F_1(s|X_j))F_1(s|X_i))} \end{aligned}$$

$AUC(s)$ definitions for control definition 1 have also been proposed. For example, the $AUC(s)$ for cumulative cases and definition 1 of controls (C1) is given by:

$$\begin{aligned} AUC_{1,C1}(s) &= P(\tilde{M}(X_i) > \tilde{M}(X_j) | D_i = 1 \text{ and } T_i \leqslant s \text{ and } T_j > s) \\ &= \frac{\mathrm{E}_{X_i,X_j}(I(\tilde{M}(X_i) > \tilde{M}(X_j))(1 - F_1(s|X_j) - F_2(s|X_j))F_1(s|X_i))}{\mathrm{E}_{X_i,X_j}((1 - F_1(s|X_j) - F_2(s|X_j))F_1(s|X_i))} \end{aligned}$$

Of note, accuracy measures using control definition 1 also depend on the cumulative distribution function of the competing event $F_2(s|X)$. Thus, such measures might be less suitable if the main goal is to assess the relevance of a marker (or a prognostic model) for predicting the absolute risk of the event of interest only but could be valuable for assessing joint models for the cumulative incidence of both competing events.

D. Proof of Lemma 3.1 and estimation of the asymptotic variance

D.1 *Consistency*

It is assumed that $\hat{G}$ is consistent for G. Thus, Slutsky's lemma shows that the weights converge in probability to

$$W_{ij,1} = G(\tilde{T}_i - |X_i)G(\tilde{T}_i|X_j) \text{ and } W_{ij,2} = G(\tilde{T}_i - |X_i)G(\tilde{T}_j - |X_j)$$

as $n \to \infty$. By the law of large numbers and Slutsky's Lemma it follows that $\hat{\mathcal{C}}_1(t)$ converges in probability to

$$\frac{\mathrm{E}_{X_i,X_j}\left[Q^{ij}(t)\left(\mathrm{E}\{\tilde{A}_{ij}\tilde{N}_i^1(t)W_{ij,1}^{-1}|X_i,X_j\} + \mathrm{E}\{\tilde{B}_{ij}\tilde{N}_i^1(t)W_{ij,2}^{-1}|X_i,X_j\}\right)\right]}{\mathrm{E}_{X_i,X_j}\left[\left(\mathrm{E}\{\tilde{A}_{ij}\tilde{N}_i^1(t)W_{ij,1}^{-1}|X_i,X_j\} + \mathrm{E}\{\tilde{B}_{ij}\tilde{N}_i^1(t)W_{ij,2}^{-1}|X_i,X_j\}\right)\right]}. \tag{D.1}$$

By applying equations (3.1) and (3.2) we have

$$\begin{aligned}\mathrm{E}(\tilde{A}_{ij}\tilde{N}_i^1(t)|X_i,X_j) &= \int_0^t \mathrm{E}(I\{\tilde{T}_j > s|X_j\})\,\mathrm{E}(\mathrm{d}\tilde{N}_i^1(s)|X_i) \\ &= \int_0^t G(s|X_j)\,S(s|X_j)\,G(s-|X_i)\mathrm{d}F_1(s|X_i)\end{aligned}$$

and hence

$$\mathrm{E}(\tilde{A}_{ij}\tilde{N}_i^1(t)W_{ij,1}^{-1}|X_i,X_j) = \int_0^t S(s|X_j)\mathrm{d}F_1(s|X_i).$$

Similarly by equation (3.2) we have

$$\begin{aligned}\mathrm{E}(\tilde{B}_{ij}\tilde{N}_i^1(t)|X_i,X_j) &= \mathrm{E}(\Delta_i\Delta_j B_{ij}N_i^1(t)|X_i,X_j) \\ &= \int_0^t\int_0^s G(v-|X_j)\,G(s-|X_i)\mathrm{d}F_2(v|X_j)\ dF_1(s|X_i)\end{aligned}$$

which yields

$$\mathrm{E}(\tilde{B}_{ij}\tilde{N}_i(t)W_{ij,2}|X_i,X_j) = \int_0^t F_2(s|X_j)\mathrm{d}F_1(s|X_i).$$

Inserting shows that expression (D.1) equals the expression for $C_1(t)$ given in (2.4):

$$\frac{\mathrm{E}_{X_i,X_j}\left[Q^{ij}(t)\int_0^t\{S(s|X_j) + F_2(s|X_j)\}\mathrm{d}F_1(s|X_i)\right]}{\mathrm{E}_{X_i,X_j}\left[\int_0^t\{S(s|X_j) + F_2(s|X_j)\}\mathrm{d}F_1(s|X_i)\right]} = \frac{\mathrm{E}_{X_i,X_j}\left[Q^{ij}(t)\int_0^t\{1 - F_1(s|X_j)\}\mathrm{d}F_1(s|X_i)\right]}{\mathrm{E}_{X_i,X_j}\left[\int_0^t\{1 - F_1(s|X_j)\}\mathrm{d}F_1(s|X_i)\right]}.$$

D.2 *Weak convergence*

If the estimate of the conditional censoring distribution converges weakly, more precisely under the following assumption:

$$\sqrt{n}\{\hat{G}(s|x) - G(s|x)\} = \frac{1}{\sqrt{n}} \sum_{i=1}^{n} IF_G(s, x; \tilde{T}_i, \tilde{D}_i, X_i) + o_p(1), \tag{D.2}$$

then the weak convergence of $\hat{\mathcal{C}}_1(t)$ follows from the i.i.d. representation

$$\sqrt{n}\{\hat{\mathcal{C}}_1(t) - \mathcal{C}_1(t)\} = \frac{1}{\sqrt{n}} \sum_{i=1}^{n} IF_{\mathcal{C}_1}(t; IF_G; \tilde{T}_i, \tilde{D}_i, X_i) + o_p(1) \tag{D.3}$$

where $IF_{\mathcal{C}_1}$ is the influence function of $\hat{\mathcal{C}}_1$ which depends on IF_G. A rigorous proof of the i.i.d representation (D.3) can be obtained with the functional delta method, alternatively it can be deduced from U-statistics theory (Van der Vaart, 1998, Chap. 12 and 20). See also Hung and Chiang (2010); Datta *and others* (2010) or Blanche *and others* (2013) for the details in a similar situation. We now derive an explicit form for the influence function in the special case where $\hat{G}$ is the marginal Kaplan-Meier estimate.

In what follows we assume independent censoring and denote $G(t)$ for the probability of being uncensored at time t, that is $G(t) = \mathrm{P}(C > t)$, and denote $S_{\tilde{T}}(t) = \mathrm{P}(\tilde{T} > t)$. In addition, we respectively denote by the shorthand notations $\mathcal{C}_1^{num}(t)$ and $\mathcal{C}_1^{den}(t)$ the numerator and the denominator of $\mathcal{C}_1(t)$ as defined at equation (2.4).

The influence function of the marginal Kaplan-Meier estimator for the marginal censoring survival function is given by

$$IF_G(t; \tilde{T}_i, \tilde{D}_i) = -G(t) \int_0^t \frac{dM_{C_i}(u)}{S_{\tilde{T}}(u)} \tag{D.4}$$

with $M_{C_i}(t) = I\{\tilde{T}_i \leqslant t, \tilde{D}_i = 0\} - \int_0^t I\{\tilde{T}_i \geqslant u\} d\Lambda_C(u)$, where $\Lambda_C(\cdot)$ is the cumulative hazard function of the censoring variable (see for instance Gill, 1994).

For all $\tilde{T}_i < \tau$ and $\tilde{T}_j < \tau$, combining first order Taylor expansions of the function $(x, y) \mapsto$

$1/(xy)$ at $(x, y) = \big(\hat{G}(\tilde{T}_i-), \hat{G}(\tilde{T}_i)\big)$ and at $\big(\hat{G}(\tilde{T}_i-), \hat{G}(\tilde{T}_j-)\big)$ with (D.2) and (D.4) yields

$$\sqrt{n}\left(\hat{W}_{ij,1}^{-1} - W_{ij,1}^{-1}\right) = \frac{1}{\sqrt{n}} \sum_{k=1}^{n} IF_{W_1}(\tilde{T}_i; \tilde{T}_k, \tilde{D}_k) + o_p(1), \tag{D.5}$$

$$\text{and} \quad \sqrt{n}\left(\hat{W}_{ij,2}^{-1} - W_{ij,2}^{-1}\right) = \frac{1}{\sqrt{n}} \sum_{k=1}^{n} IF_{W_2}(\tilde{T}_i, \tilde{T}_j; \tilde{T}_k, \tilde{D}_k) + o_p(1), \tag{D.6}$$

when defining

$$IF_{W_1}(\tilde{T}_i; \tilde{T}_k, \tilde{D}_k) = 2W_{ij,1}^{-1} \int_0^{\tilde{T}_i} \frac{dM_{C_k}(u)}{S_{\tilde{T}}(u)}, \tag{D.7}$$

$$\text{and} \quad IF_{W_2}(\tilde{T}_i, \tilde{T}_j; \tilde{T}_k, \tilde{D}_k) = W_{ij,2}^{-1} \left\{ \int_0^{\tilde{T}_i} \frac{dM_{C_k}(u)}{S_{\tilde{T}}(u)} + \int_0^{\tilde{T}_j} \frac{dM_{C_k}(u)}{S_{\tilde{T}}(u)} \right\}, \tag{D.8}$$

with the notations $W_{ij,1} = G(\tilde{T}_i-)G(\tilde{T}_i)$ and $W_{ij,2} = G(\tilde{T}_i-)G(\tilde{T}_j-)$. Note that $\hat{W}_{ij,1}^{-1}$ and so its influence function does no longer depend on observation from subject j when assuming the censoring independent of the covariates. In addition, let us respectively denote the numerator and denominator of $\hat{\mathcal{C}}_1(t)$ defined at equation (3.4) divided by n^2 by $\hat{\mathcal{C}}_1^{num}(t)$ and $\hat{\mathcal{C}}_1^{den}(t)$. As a consequence of (D.5),(D.6),(D.7), (D.8) and of the expression of the numerator of (3.4) it follows

$$\begin{aligned}\sqrt{n}\left(\hat{\mathcal{C}}_1^{num}(t) - \mathcal{C}_1^{num}(t)\right) = \frac{\sqrt{n}}{n^3} \sum_{i=1}^{n}\sum_{j=1}^{n}\sum_{k=1}^{n} \Bigg[\Bigg\{ & \tilde{A}_{ij}\left\{W_{ij,1}^{-1} + IF_{W_1}(\tilde{T}_i; \tilde{T}_k, \tilde{D}_k)\right\} \\ & + \tilde{B}_{ij}\left\{W_{ij,2}^{-1} + IF_{W_2}(\tilde{T}_i, \tilde{T}_j; \tilde{T}_k, \tilde{D}_k)\right\} \\ & \Bigg\}\tilde{N}_i^1(t)Q_{ij} - \mathcal{C}_1^{num}(t)\Bigg] + o_p(1).\end{aligned} \tag{D.9}$$

By the same arguments, a similar result holds for $\sqrt{n}\left(\hat{\mathcal{C}}_1^{den}(t) - \mathcal{C}_1^{den}(t)\right)$: the decomposition is the same than the one of the right term of equation (D.9) except that $\mathcal{C}_1^{num}(t)$ is replaced by $\mathcal{C}_1^{den}(t)$ and that the term Q_{ij} disappears. For all $t < \tau$, a first order Taylor expansion of $(x, y) \mapsto x/y$ at $(x, y) = \big(\hat{\mathcal{C}}_1^{num}(t), \hat{\mathcal{C}}_1^{den}(t)\big)$ further leads to:

$$\sqrt{n}\left(\hat{\mathcal{C}}_1(t) - \mathcal{C}_1(t)\right) = \frac{\sqrt{n}}{n^3} \sum_{i=1}^{n}\sum_{j=1}^{n}\sum_{k=1}^{n} \psi_{ijk}(t) + o_p(1)$$

where

$$\psi_{ijk}(t) = \Bigg\{ \tilde{A}_{ij} W_{ij,1}^{-1} \left(1 + 2\int_0^{\tilde{T}_i} \frac{dM_{C_k}(u)}{S_{\tilde{T}}(u)} \right) + \tilde{B}_{ij} W_{ij,2}^{-1} \left(1 + \int_0^{\tilde{T}_i} \frac{dM_{C_k}(u)}{S_{\tilde{T}}(u)} + \int_0^{\tilde{T}_j} \frac{dM_{C_k}(u)}{S_{\tilde{T}}(u)} \right) \Bigg\} \tilde{N}_i^1(t) \Big(Q_{ij} - \mathcal{C}_1(t) \Big) \Big/ \mathcal{C}_1^{den}(t)$$

Finally, we use the Hájek projection principle and U-statistic theory (Van der Vaart, 1998, Sec. 11.3 & Chap. 12) to write the i.i.d decomposition (D.3). More precisely, we first define the symmetric kernel $h_{ijk}(t) = \big\{\psi_{ijk}(t) + \psi_{jik}(t) + \psi_{jki}(t)\big\}/3!$ following page 171 of Serfling (1980) before applying Theorem 12.3 of Van der Vaart (1998) to it. It therefore follows:

$$\sqrt{n}\left(\hat{\mathcal{C}}_1(t) - \mathcal{C}_1(t)\right) = \frac{1}{\sqrt{n}} \sum_{i=1}^{n} IF_{\mathcal{C}_1}(t; \tilde{T}_i, \tilde{D}_i, X_i) + o_p(1)$$

where $IF_{\mathcal{C}_1}(t; \tilde{T}_i, \tilde{D}_i, X_i) = \mathbb{E}\Big[\psi_{ijk}(t) + \psi_{jik}(t) + \psi_{jki}(t) \Big| (\tilde{T}_i, \tilde{D}_i, X_i)\Big]$.

D.3 *Consistent estimation of the standard error of $\hat{\mathcal{C}}_1$*

Let us denote $\hat{M}_{C_k}(t) = I\{\tilde{T}_k \leqslant t, \tilde{D}_k = 0\} - \int_0^t I\{\tilde{T}_k \geqslant u\} d\hat{\Lambda}_C(u)$ with $\hat{\Lambda}_C(\cdot)$ the usual Nelson-Aalen estimator of the cumulative hazard function of the censoring variable C, and let $\hat{S}_{\tilde{T}}(\cdot)$ be the empirical estimator of $S_{\tilde{T}}(\cdot)$. By defining a plug-in estimate $\hat{\psi}_{ijk}(t)$ and using empirical means for conditional expectation estimation, we can consistently estimate $IF_{\mathcal{C}_1}(t; \tilde{T}_i, \tilde{D}_i, X_i)$ by

$$\widehat{IF}_{\mathcal{C}_1}(t; \tilde{T}_i, \tilde{D}_i, X_i) = \frac{1}{n^2} \sum_{j=1}^{n} \sum_{k=1}^{n} \left\{ \hat{\psi}_{ijk}(t) + \hat{\psi}_{jik}(t) + \hat{\psi}_{jki}(t) \right\}$$

where

$$\hat{\psi}_{ijk}(t) = \Bigg\{ \tilde{A}_{ij} \hat{W}_{ij,1}^{-1} \left(1 + 2\int_0^{\tilde{T}_i} \frac{d\hat{M}_{C_k}(u)}{\hat{S}_{\tilde{T}}(u)} \right) + \tilde{B}_{ij} \hat{W}_{ij,2}^{-1} \left(1 + \int_0^{\tilde{T}_i} \frac{d\hat{M}_{C_k}(u)}{\hat{S}_{\tilde{T}}(u)} + \int_0^{\tilde{T}_j} \frac{d\hat{M}_{C_k}(u)}{\hat{S}_{\tilde{T}}(u)} \right) \Bigg\} \tilde{N}_i^1(t) \Big(Q_{ij} - \hat{\mathcal{C}}_1(t) \Big) \Big/ \hat{\mathcal{C}}_1^{den}(t).$$

A consistent estimator of the asymptotic variance of $\hat{\mathcal{C}}_1(t)$ is therefore defined by

$$\hat{\sigma}^2_{\mathcal{C}_1(t)} = \frac{1}{n} \sum_{i=1}^{n} \left\{ \widehat{IF}_{\mathcal{C}_1}(t; \tilde{T}_i, \tilde{D}_i, X_i) \right\}^2.$$

E. Simulation results for truncation at the median

Table 1. Average bias and root mean square error (RMSE) for 3 different estimators of $\mathcal{C}_1(t)$ averaged over 1000 data sets simulated under the 2 scenarios CR1 and CR2 for varying sample size N, independent ($\gamma_1 = 0$) or covariate dependent censoring ($\gamma_1 = 1$), respectively, and varying censoring rates. t was chosen as the median of the marginal time-to-event distribution. Column 3 shows the expected proportion of right-censored event times amongst observations with $\tilde{T} < t$. Columns 4-6 show average bias (RMSE) for the three estimators (multiplied by 100 for easier readability).

N	γ_1	Censored before t (%)	$\hat{\mathcal{C}}_{1,\text{naive}}(t)$	$\hat{\mathcal{C}}_{1,\text{KM}}(t)$	$\hat{\mathcal{C}}_{1,\text{Cox}}(t)$
CR1: $\mathcal{C}_1(t) = 67.6\%, \beta_1 = 1, \beta_2 = 1, \lambda_{01} = 1, \lambda_{02} = 2$, t= median					
250	0	25	0.6(4.4)	0.1(4.3)	0.1(4.3)
250	0	50	1.5(5.2)	0.2(5.1)	0.2(5.1)
250	0	75	3.4(7.1)	0.4(7.9)	0.4(7.8)
250	1	25	0.2(4.6)	−0.3(4.6)	0.0(4.6)
250	1	50	0.6(5.8)	−0.6(5.9)	−0.7(6.9)
250	1	75	1.5(8.1)	−0.9(9.2)	−3.9(12.3)
1000	0	25	0.4(2.1)	−0.1(2.0)	−0.1(2.0)
1000	0	50	1.4(2.7)	−0.1(2.5)	−0.1(2.5)
1000	0	75	3.2(4.4)	−0.1(3.7)	−0.1(3.7)
1000	1	25	0.0(2.2)	−0.6(2.3)	−0.2(2.3)
1000	1	50	0.4(2.8)	−1.0(3.0)	−0.6(4.1)
1000	1	75	1.5(4.0)	−1.6(4.5)	−2.9(8.4)
CR2: $\mathcal{C}_1(t) = 85.8\%, \beta_1 = 2, \beta_2 = -1, \lambda_{01} = 1, \lambda_{02} = 0.5$, t= median					
250	0	25	0.5(1.9)	0.0(1.9)	0.0(1.9)
250	0	50	1.1(2.3)	0.0(2.2)	0.0(2.2)
250	0	75	2.1(3.3)	0.2(3.6)	0.3(3.5)
250	1	25	0.4(2.0)	−0.1(2.0)	0.0(1.9)
250	1	50	0.9(2.6)	−0.2(2.7)	−0.1(2.6)
250	1	75	1.6(3.8)	−0.5(4.4)	−1.1(5.5)
1000	0	25	0.5(1.0)	0.0(0.9)	0.0(0.9)
1000	0	50	1.1(1.5)	0.0(1.1)	0.0(1.1)
1000	0	75	2.0(2.3)	−0.1(1.7)	−0.1(1.7)
1000	1	25	0.4(1.1)	−0.1(1.0)	0.0(0.9)
1000	1	50	0.9(1.5)	−0.3(1.3)	−0.1(1.3)
1000	1	75	1.5(2.2)	−0.6(2.3)	−1.0(4.6)

Table 2. Coverage of confidence intervals for the same simulation scenarios as in Table 1. Columns 8-10 display observed coverage of 95% percentile bootstrap confidence intervals for all 3 estimators, column 7 shows coverage of asymptotic Wald-type confidence intervals for $\hat{\mathcal{C}}_{1,\mathrm{KM}}(t)$. Columns 4-6 show the empirical standard error for the 1000 estimates and the average asymptotic and bootstrap standard errors of $\hat{\mathcal{C}}_{1,\mathrm{KM}}(t)$.

			Std.error KM			Coverage			
N	γ_1	Censored before t (%)	empirical	average asymptotic	average bootstrap	asymptotic KM	bootstrap naive	bootstrap KM	bootstrap Cox
			CR1: $\mathcal{C}_1(t) = 67.6\%, \beta_1 = 1, \beta_2 = 1, \lambda_{01} = 1, \lambda_{02} = 2$, t= median						
250	0	25	0.0434	0.0433	0.0435	93.3	93.1	93.3	93.2
250	0	50	0.0506	0.0496	0.0502	93.1	91.5	93.3	93.1
250	0	75	0.0785	0.068	0.0713	88.7	86.6	90.5	90.1
250	1	25	0.0456	0.0452	0.0459	93.6	94.5	93.9	94.1
250	1	50	0.0592	0.0547	0.0561	92.3	91.8	92.8	92.1
250	1	75	0.0912	0.0752	0.0807	87.6	89.1	89.5	86.8
1000	0	25	0.0204	0.0217	0.0215	94.9	95.8	94.8	94.9
1000	0	50	0.0245	0.025	0.0249	95.0	90.9	94.9	94.8
1000	0	75	0.0373	0.0373	0.0374	94.5	82.0	94.5	94.5
1000	1	25	0.0222	0.0227	0.0227	94.6	95.4	94.7	94.8
1000	1	50	0.0279	0.0278	0.0279	94.1	94.7	94.4	91.2
1000	1	75	0.0421	0.0405	0.041	92.8	92.1	93.3	83.0
			CR2: $\mathcal{C}_1(t) = 85.8\%, \beta_1 = 2, \beta_2 = -1, \lambda_{01} = 1, \lambda_{02} = 0.5$, t= median						
250	0	25	0.0189	0.0195	0.0196	95.2	93.7	95.1	95.0
250	0	50	0.0219	0.0223	0.0225	93.6	88.4	94.1	94.2
250	0	75	0.036	0.0324	0.0337	89.1	79.9	90.1	89.0
250	1	25	0.0202	0.0209	0.0212	95.1	93.3	95.7	95.1
250	1	50	0.0265	0.0265	0.0271	95.0	90.2	95.4	95.5
250	1	75	0.044	0.0399	0.0413	90.2	84.6	91.7	95.4
1000	0	25	0.00922	0.0097	0.00966	95.6	91.9	95.4	95.3
1000	0	50	0.0106	0.011	0.011	95.5	80.0	95.3	95.1
1000	0	75	0.017	0.0167	0.0168	94.4	61.7	94.5	94.2
1000	1	25	0.00996	0.0104	0.0104	95.2	92.6	95.1	95.3
1000	1	50	0.0128	0.0132	0.0133	95.2	86.4	95.4	95.7
1000	1	75	0.0218	0.021	0.0212	94.1	81.1	94.4	93.1

References

Bender, R., Augustin, T. and Blettner, M. (2005). Generating survival times to simulate Cox proportional hazards models. *Statistics in Medicine* **24**, 1713–1723.

Beyersmann, J., Dettenkofer, M., Bertz, H. and Schumacher, M. (2007). A competing risks analysis of bloodstream infection after stem-cell transplantation using subdistribution hazards and cause-specific hazards. *Statistics in Medicine* **26**, 5360–5369.

Blanche, P., Dartigues, J-F. and Jacqmin-Gadda, H. (2013). Estimating and comparing time-dependent areas under receiver operating characteristic curves for censored event times with competing risks. *Statistics in Medicine* **32**, 5381–5397.

Datta, S., Bandyopadhyay, D. and Satten, G.A. (2010). Inverse probability of censoring weighted u-statistics for right-censored data with an application to testing hypotheses. *Scandinavian Journal of Statistics* **37**, 680–700.

Fine, J. P. and Gray, R. J. (1999). A proportional hazards model for the subdistribution of a competing risk. *Journal of the American Statistical Association* **446**, 496–509.

Gill, R. D. (1994). Lectures on survival analysis. In: *Bakry, Dominique (ed.) et al., Lectures on probability theory. Ecole d'Eté de Probabilités de Saint-Flour XXII-1992. Summer School, 9th- 25th July, 1992, Saint-Flour, France. Berlin: Springer-Verlag. Lect. Notes Math. 1581, 115-241* .

Heagerty, P. J. and Zheng, Y. (2005). Survival model predictive accuracy and ROC curves. *Biometrics* **61**, 92–105.

Hung, H. and Chiang, C.T. (2010). Estimation methods for time-dependent AUC models with survival data. *Canadian Journal of Statistics* **38**, 8–26.

Koller, M. T., Raatz, H., Steyerberg, E. W. and Wolbers, M. (2012). Competing risks and the clinical community: irrelevance or ignorance? *Statistics in Medicine* **31**, 1089–1097.

Mogensen, U. B., Ishwaran, H. and Gerds, T. A. (2012). Evaluating random forests for survival analysis using prediction error curves. *Journal of Statistical Software* **50**, 1–23.

Saha, P. and Heagerty, P. J. (2010). Time-dependent predictive accuracy in the presence of competing risks. *Biometrics* **66**, 999–1011.

Serfling, R.J. (1980). *Approximation theorems of mathematical statistics*. John Wiley & Sons New York.

Van der Vaart, A. W. (1998). *Asymptotic Statistics*. Cambridge University Press.

Zheng, Y., Cai, T., Jin, Y. and Feng, Z. (2012). Evaluating prognostic accuracy of biomarkers under competing risk. *Biometrics* **68**, 388–396.
